# Cutaneous Adverse Effects in Patients Treated with BTK Inhibitors

**DOI:** 10.3390/cancers18030371

**Published:** 2026-01-24

**Authors:** Ewa Robak, Tadeusz Robak

**Affiliations:** 1 Department of Dermatology, Medical University of Lodz, 90-647 Łódź, Poland; ewa.robak@umed.lodz.pl; 2 Department of Hematology, Medical University of Lodz, 93-510 Lodz, Poland; 3 Department of General Hematology and Internal Medicine, Copernicus Memorial Hospital, 93-510 Lodz, Poland

**Keywords:** acalabrutinib, bleeding, BTK inhibitors, ibrutinib, infections, mucosal symptoms, neutrophilic dermatoses, pirtobrutinib, rash, skin toxicity, zanubrutinib

## Abstract

Bruton’s tyrosine kinase (BTK) inhibitors have revolutionized the treatment landscape for patients with indolent lymphoid malignancies such as chronic lymphocytic leukemia (CLL) and mantle cell lymphoma (MCL). The most common adverse events include cardiac arrhythmia, bleeding, infection, diarrhea, arthralgias, hypertension, and skin changes. Second-generation BTK inhibitors, e.g., acalabrutinib and zanubrutinib and the non-covalent BTK inhibitor pirtobrutinib, are less toxic than the first-generation BTK inhibitor ibrutinib. The most common toxic skin symptoms related to BTKi treatment include hemorrhage, bleeding events, bruising, skin ecchymoses, and contusion; they are particularly common in patients treated with ibrutinib. Other dermatologic symptoms include rash, cellulitis, skin infections, subcutaneous abscesses and peripheral edema. This article discusses the development of skin symptoms in patients with ibrutinib and newer BTK inhibitors, and summarizes their clinical and pathological characteristics. A literature search was performed using PubMed, Web of Science, and Google Scholar for articles published in English. Additional relevant publications were obtained by reviewing the references from the chosen articles.

## Simple Summary

Patients treated with BTK inhibitors frequently demonstrate dermatologic toxicities, such as bruising, rash, infections, mucosal symptoms, neutrophilic dermatoses, vasculitis, infections, nail plate abnormalities and hair changes. In most cases, skin toxicities remain mild. Proactive management is crucial in order to limit dose-intensity modification. For most patients, these skin changes are benign and do not require treatment: discontinuation or topical or oral corticosteroid use should be considered until cutaneous symptoms disappear. However, in some patients, BTKi dose reduction, treatment interruption, or even cessation of treatment is recommended.

## 1. Introduction

Over the past decade, the treatment landscape for indolent B-cell lymphoid malignancies has shifted from chemotherapy and immunochemotherapy to targeted oral therapies [1], with the Bruton’s tyrosine kinase (BTK) inhibitors ibrutinib, acalabrutinib, and zanubrutinib playing the key role [2,3]. These agents effectively inhibit B-cell receptor (BCR) signalling in neoplastic B-cells and they are most commonly used in slow-growing indolent non-Hodgkin lymphoma (NHL). According to the 5th Edition of the World Health Organization Classification and International Consensus Classification (ICC) indolent NHLs comprise chronic lymphocytic leukemia/small lymphocytic lymphoma (CLL/SLL), follicular lymphoma (FL), marginal zone lymphoma (MZL), lymphoplasmacytic lymphoma, mantle cell lymphoma (MCL) and hairy cell leukemia (HCL), together with various other rarer entities [4]. BTK inhibitors can also play active roles in treating autoimmune disorders like autoimmune haemolytic anemia (AIHA), immune thrombocytopenia (IT), multiple sclerosis, pemphigus vulgaris, atopic dermatitis, rheumatoid arthritis, systemic lupus erythematosus, and Sjögren’s disease [5].

BTK inhibitors are divided into covalent, irreversible BTKis, such as ibrutinib, acalabrutinib, and zanubrutinib, and non-covalent, reversible BTK inhibitors, such as pirtobrutinib and nemtabrutinib. Ibrutinib was the first-in-class covalent, irreversible BTK inhibitor, which was approved in 2013 for the treatment of CLL [1]. Since then, two other covalent, irreversible, second-generation BTK inhibitors, acalabrutinib and zanubrutinib, were developed and approved. These next-generation BTK inhibitors present more selective binding to BTK than ibrutinib and hence possess a better safety profile [6]. However, they are still associated with similar dermatological toxicities [7,8,9,10,11,12].

Of the non-covalent, reversible BTK inhibitors, the most advanced forms tested in clinical trials are pirtobrutinib and nemtabrutinib, which overcome drug resistance against covalent BTK inhibitors. Both agents are selective BTK inhibitors, with low off-target activity and low risk of hematologic and cardiac toxicity [6,7,8] and as neither bind to the C481 residue, both are effective in patients with BTK C481 mutation. While BTK inhibitors are relatively safe and well-tolerated drugs, their use has been associated with certain common adverse events (AEs) [1,3]. The most common include cardiac arrhythmia, bleeding, infection, diarrhea, arthralgias, hypertension, and skin changes [9]. Cutaneous toxicity has been reported as one of the most common non-hematological side effects of ibrutinib [9].

The most common cutaneous AEs associated with BTK inhibitors include rash, bleeding and bruising, ecchymoses, neutrophilic panniculitis, hair and nail changes, skin infections, bruising, petechiae, and purpuric eruption [12,13,14,15,16,17]. Dermatological AEs have been reported in 2–27% of patients treated with ibrutinib; most were mild to moderate intensity [10,11,12] and commonly occurred during the first year of treatment [12]. While dermatological AEs are generally scarce, their presence can lead to BTKi discontinuation in a small proportion of patients [12,13,14,15]. A retrospective analysis of 46 real-world acalabrutinib-treated CLL patients intolerant to ibrutinib found that rash led to discontinuation of acalabrutinib in 10 (22%) patients. During treatment with acalabrutinib, with a median follow-up of five months, a rash was noted in five (7%) patients [15]. In a direct comparison of acalabrutinib with ibrutinib in relapsed/refractory (R/R) CLL patients, peripheral edema (any grade) was observed in 9.8% of the acalabrutinib group and in 14.4% of the ibrutinib group [10]. Rash was observed in 9.8% and 12.5% of the group, respectively.

Dermatological toxicities have been reported in ≥10% patients treated with zanubrutinib [18]. Grade 4 dermatological toxicities developed within one month after treatment; these included bruising, maculopapular rash, ecchymosis, haemorrhagic blister, acne-like rash, papulopustular rash, and skin infections. A direct comparison of zanubrutinib with ibrutinib in R/R CLL patients as part of a randomized study [11] identified any-grade hemorrhage in 42.3% of the zanubrutinib group and 41.4% of the ibrutinib group. Major hemorrhages also occurred at similar frequencies in the two arms (3.7% and 4.3%, respectively).

The only ncBTKi approved for the treatment of lymphoid malignancies is pirtobrutinib, which has demonstrated similar dermatologic toxicities to ibrutinib in a direct comparison [8]. Several clinical trials indicated that BTKi-related skin side effects are well tolerated and can be managed with symptomatic treatment in most patients; however, in some patients, drug discontinuation is required [12,19].

The mechanism of skin toxicity in patients treated with BTK inhibitors is multifactorial and mostly related to the off-target activity of these agents. In addition to BTK inhibition, ibrutinib can inhibit other kinases, including Src (rous sarcoma virus) kinase, tyrosine-protein kinase (TEC), and epidermal growth factor receptor (EGFR) [20]. In contrast, second-generation BTK inhibitors, e.g., acalabrutinib and zanubrutinib, have fewer off-target effects. Some of these effects are associated with the epidermal growth factor receptor (EGFR) pathway, which stimulates epidermal growth by regulating the normal growth and differentiation process of the epidermis. EGFRs are expressed on keratinocytes, which are distributed in the basal and suprabasal layers of the epidermis. EGFR inhibition causes premature skin cell differentiation, inflammation, apoptosis, skin atrophy, telangiectasia, and photosensitivity [21]. In addition, in the first few weeks of BTKi therapy in patients with CLL and the leukemic form of non-Hodgkin lymphoma (NHL), the patients may demonstrate inhibition of c-kit and platelet-derived growth factor (PDGF) receptors resulting in increased lymphocytosis.

Dermatological toxicities associated with BTK inhibitors are mainly noted in the first year of treatment, and their incidence decreases gradually with time [12]. Treatment of BTKi-induced dermatologic toxicity depends on the grade of EAs. In patients with moderate to severe dermatological toxicities, topical steroid ointments used alone or in combination with topical emollients are recommended. In patients with grade 4 toxicities, systemic antibiotics or antibiotic ointments should be used in patients with infection; otherwise dose reduction or temporarily discontinuation should be considered [19,22].

This article summarizes the clinical and pathological characteristics of abnormalities of the skin and mucosal symptoms, nail lesions, and hair in patients treated with BTK inhibitors. The literature included in the review was identified through a search of PubMed, Web of Science, and Google Scholar; all articles were published in English. Additional relevant publications were obtained by reviewing the references from the chosen articles.

## 2. Hemorrhagic Symptoms in the Skin

**Figure 1:**
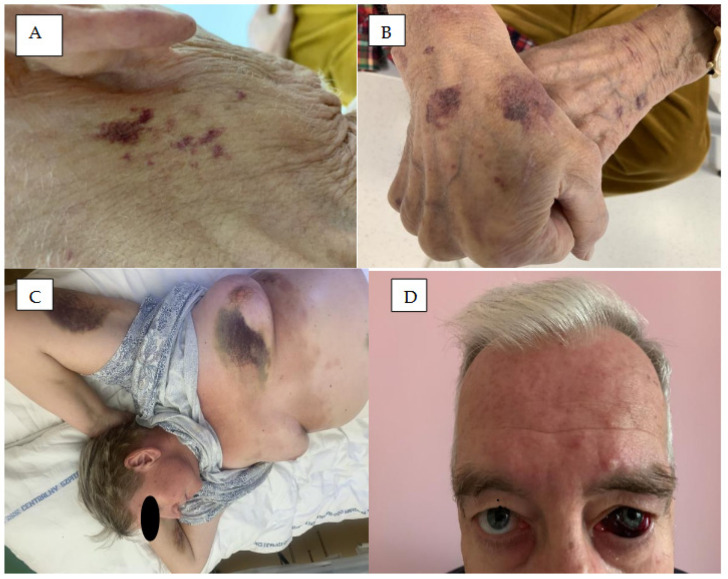
Hemorrhagic skin complications of BTK inhibitors. (**A**) Bruising in the neck of CLL patient treated with ibrutinib. (**B**) Bruising in the back of the hands of CLL patient treated with acalabrutinib. (**C**) Skin hemorrhages on the trunk and arm of MCL patient treated with ibrutinib. (**D**) Hematoma of the left eye in a patient on ibrutinib.

**Figure 2:**
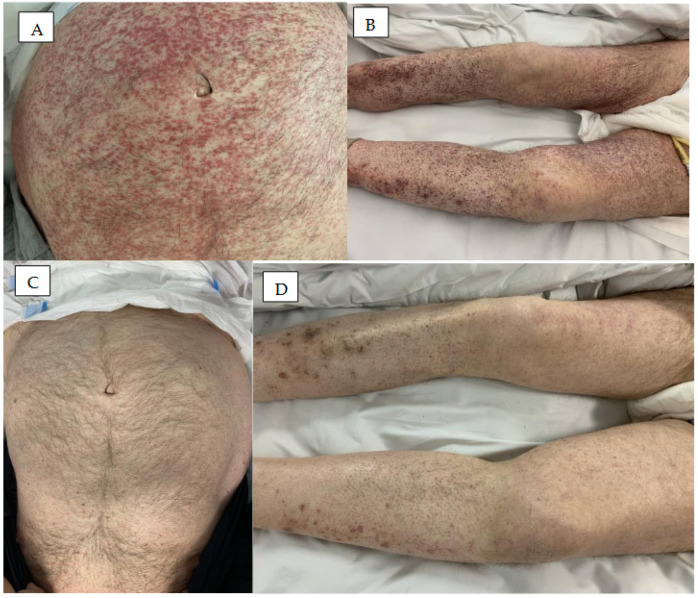
Petechiae in a patient with MCL emerged two weeks post initiation of ibrutinib treatment (**A**,**B**) and either vanished or considerably diminished two weeks after ceasing ibrutinib (**C**,**D**).

The common hemorrhagic skin complications of BTK inhibitors are bruising, hematomas, and petechiae (Figure 1 and Figure 2). Severe bleeding events have been observed in 1–9% of patients treated with ibrutinib, 1–3% of those treated with acalabrutinib, and 2.5% of those treated with zanubrutinib [12]. An analysis of 15 ibrutinib clinical trials for lymphoid malignancies found any-grade bleeding to occur in 40% of patients [23], with only 4% of patients developing major hemorrhage, and 1% of all treated cases leading to ibrutinib discontinuation. In a pooled analysis of three pivotal studies encompassing 330 patients with CLL treated with ibrutinib, bleeding/bruising events were observed in 55% of patients, and 25 major hemorrhage events were reported in 21 patients (6%) [24].

The haemorrhagic skin complications of BTK inhibitors include bruising, skin hematomas, ecchymoses, haemorrhagic crusting or blisters, and purpuric nodules/eruption [24,25,26]. The risk of BTK inhibitor-associated bleeding peaks during the first year of treatment and decreases over subsequent years.

The elevated risk of bleeding is mainly due to inhibition of platelet function by BTK inhibitors, largely attributed to interference with platelet glycoprotein (GP) signalling and TEC inhibition involved in platelet activation and aggregation [27]. Moreover, patients treated with BTKis show reduced collagen-mediated platelet aggregation, which correlates with the occurrence of clinical bleeding [28]. In addition, patients treated with dual-antiplatelet therapy or systemic anticoagulants are at a higher risk of bleeding following BTK inhibitor use [29,30]. In addition, patients treated with ibrutinib and an anticoagulant have demonstrated a 2.5-fold higher risk of bleeding compared with those treated with ibrutinib alone [31].

Petechial rash is believed to be caused by BTK-mediated platelet dysfunction and usually develops several months after treatment initiation. Edematous, or purpuric papular rashes, appear in the first few weeks of treatment and have been associated with an immune-mediated drug reaction; they are more common in patients with a history of drug hypersensitivity [22]. Acalabrutinib and zanubrutinib are more selective BTK inhibitors with less off-target activity than ibrutinib and lower antiplatelet activity [10,11,32]. Direct comparisons between the drugs indicate acalabrutinib causes fewer hemorrhages than ibrutinib, with no such difference between zanubrutinib and ibrutinib [10,11].

A pooled analysis of 1040 patients with mature B-cell lymphoid malignancies treated with acalabrutinib monotherapy found hemorrhage to occur in 46% of cases, with 4% demonstrating a major hemorrhage (grade ≥ 3 AEs) [33]. Another pooled analysis of 779 patients treated with zanubrutinib monotherapy identified hemorrhage in 55%, including 4% major hemorrhages [18]. One case of zanubrutinib-induced petechial ecchymotic reaction has been reported in a patient with CLL [34]. In addition, Zhou et al. [35], describe the development of progressive rash in a male patient receiving zanubrutinib for two months. The patient experienced scattered papules with mild itching in both lower limbs; these papules ruptured in multiple locations and the ulcers gradually increased in size and depth. The ulcers were surrounded by erythema and exudate, with a scattered distribution and Cryptococcal infection. Zanubrutinib treatment was stopped and replaced with fluconazole for 10 months. The skin ulcers on both lower limbs disappeared.

The non-covalent reversible BTK inhibitor pirtobrutinib demonstrates improved tolerability in comparison with covalent BTK inhibitors and may provide patients with another opportunity to safely receive BTK inhibitor therapy. In a post hoc analysis of bleeding risk in patients with B-cell malignancies treated with pirtobrutinib, bleeding/bruising events were mostly low-grade, with grade 3 events occurring in fewer than 3% of patients [36]. All-grade bleeding/bruising was observed in 44.9% of patients and grade ≥ 3 in 2.8%; among these, bruising was observed in 27.8%, contusion in 22.7% and petechiae in 3.7%. Moreover, patients who received concomitant antithrombotic therapy were more likely to demonstrate bleeding/bruising events (44.9%) than those who did not (32.5%), although the antithrombotic therapy group did not experience any grade 4–5 bleeding/bruising events. A direct comparison of pirtobrutinib with ibrutinib in TN and R/R CLL patients identified a similar frequency of bleeding events between the two groups [8]. Any-grade bleeding events occurred in 34.8% of the pirtobrutinib group and 36.3% of the ibrutinib group, with grade ≥ 3 bleeding events noted in 3.3% and 2.8% of the respective groups. Any-grade bruising was noted in 13.6% of the pirtobrutinib group and 12.0% of the ibrutinib group. Petechiae and purpura were noted in 5.2% and 7.7%, respectively.

Ecchymoses or bruises have been widely described with BTK inhibitors [12,34,37,38]. In one study, contusion, petechial, and ecchymosis were observed in 23–33% of patients treated with ibrutinib, 31–39% of those receiving acalabrutinib and 43% of those receiving zanubrutinib [12]. Ecchymoses are more common in older patients, particularly on skin exposed to the sun. Among patients receiving BTK inhibitors, ecchymoses and bruises should be managed with emollients and by preventing sun exposure. However, more severe dermatologic lesions require BTKi treatment cessation. Acalabrutinib-induced ecchymotic lesions have also been reported. Kucharik et al. describe an ecchymotic patch diagnosed as cutaneous collagenous vasculopathy (CCV) on the dorsal aspect of the left forearm in a CLL patient treated with acalabrutinib; the observed skin changes were probably due to endothelial proliferations, which may be the underlying cause of the associated vascular leakage [39]. Following acalabrutinib cessation, these skin changes improved and the patch began to resolve; however, the ecchymotic patch reappeared after restarting acalabrutinib.

Guenther et al. present another patient who developed an extensive ecchymotic patch following treatment with acalabrutinib [40]. Skin changes were characterized by superficial vascular ectasia and chronic thrombotic changes with partial occlusion of the vessels by proteinaceous debris; these were located on the dorsal aspect of the left forearm. Resolution was observed six weeks after treatment discontinuation. In another patient treated with acalabrutinib, Truong et al. described extensive, indurated, confluent purpuric eruption associated with subcutaneous swelling and pain confined to both upper limbs [41]. These changes were not reduced following acalabrutinib dose reduction to 100 mg daily.

Zanubrutinib, another second-generation BTK inhibitor, can also induce eccchymotic lesions [34,37,38]. In one case, skin lesions were located on the hands and forearms and arms; the changes were characterized as purpuric, purplish, non-infiltrated vascular patches located mainly on photo-exposed areas. Another case of zanubrutinib-induced petechial ecchymotic reaction was also noted in a patient with CLL [34]. The lesions were characterized as well-demarcated, yellow-brown macular discoloration over the right breast; they extended onto the intermammary skin and the upper abdomen with petechiae scattered throughout. Their location was restricted to a previously irradiated area. In this region, histopathologic examination was consistent with ecchymosis, characterized by mild perivascular inflammation and focal erythrocyte extravasation in the papillary dermis. Skin changes were benign and did not require treatment discontinuation.

## 3. Skin Rash

**Figure 3:**
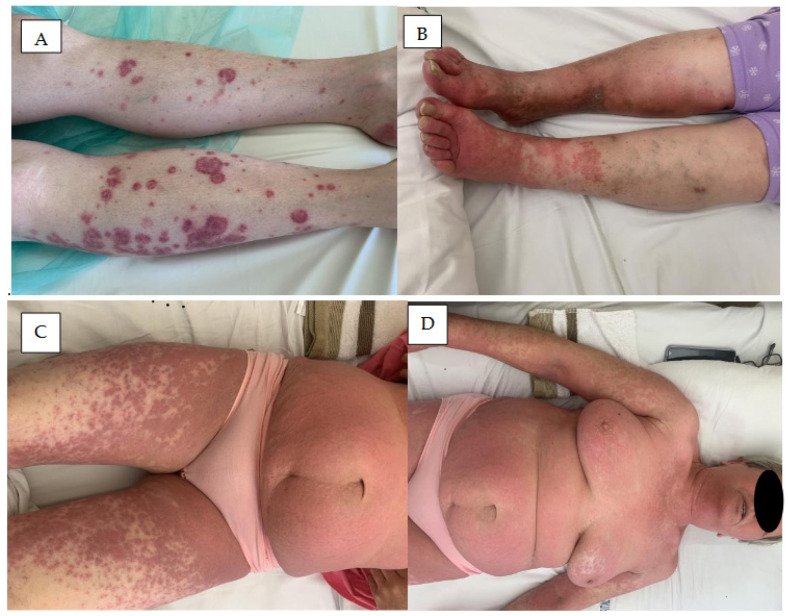
Rash in patients treated with BTK inhibitors. (**A**) Erythema multiforme on the skin of the lower limbs in a patient treated with ibrutinib for five weeks. (**B**) Erythema with vascular reaction on the feet and lower legs after three months of treatment with zanubrutinib. (**C**) Acute allergic-toxic erythema with a hemorrhagic component on the lower limbs after three weeks of treatment with ibrutinib. (**D**) Acute allergic erythema on the skin of the face and trunk in a patient treated for two weeks with ibrutinib.

BTKi-induced rashes have relatively non-specific and varied clinical presentations [10], ranging from asymptomatic ecchymosis and non-palpable petechial rash, to leukocytoclastic vasculitis-like palpable purpura (Figure 3) [12,42]. Rash has been reported in 13% to 27% of patients treated with ibrutinib, 15–18% of those treated with acalabrutinib, and 13% to 18% of those treated with zanubrutinib [12,42,43,44]. Nocco et al. identified five different types of rash morphology associated with ibrutinib therapy: non-palpable petechial rash, leukocytoclastic vasculitis-like pruritic palpable purpura, pityriasis roseae-like rash, papulopustular rash, and painless non-pruritic oedematous papules [22]. Among these, the most common clinical manifestation is acne-like rash with erythematous papules or pustules centred on the hair follicles.

The most likely mechanism of BTKi-induced skin rash is the inhibition of EGFR. However, it has also been proposed that ibrutinib-induced drug eruption may occur though the inhibition of c-kit and PDGF receptors [43]. Palpable rashes are typically pruritic and are associated with EGF receptor inhibition and inflammatory cell infiltration [43]. They have been associated with second-generation BTK inhibitor treatment, despite its higher selectivity [45]. Grade 4 skin rash was also reported in a patient treated with zanubtutinib [19].

Treatment has been associated with pityriasis rosacea-like rashes, which can develop as violaceous, scaly, pruritic plaques or papules and are typically observed on the trunk. Such rashes are most likely caused by off-target inhibition of the c-kit and PDGF receptor. In addition, off-target inhibition of EGFR can cause a papulopustular rash, which presents in the first few weeks of treatment, and can be associated with photosensitivity [17].

Ibrutinib-induced rashes have been identified in the course of B-cell lymphoid malignancies, with some patients experiencing symptoms as late as 300–400 days after beginning treatment [46]. In a study of 33 ibrutinib-intolerant patients who were subsequently treated with acalabrutinib, eight (24%) reported the occurrence of a rash; of these, 6% were rash grade ≥ 3. Of the eight recorded rash events, after acalabrutinib treatment, the rash did not recur in five patients, returned at a lower grade in one patient, and returned at the same grade in two [47].

Patients with grade 3 rashes have also demonstrated severe allergic drug reactions, such as lip tingling and tongue swelling, following ibrutinib treatment [48].

Rash lesions attributable to BTK inhibitors usually resolve over a few weeks. In one study, rash resolution was observed after a median three-week course of topical corticosteroids and oral antihistamines, without the need for BTK-inhibitor cessation [43]. Elsewhere, resolution was achieved in two weeks in patients treated with oral steroids, antihistamines and temporary BTK-inhibitor cessation [44]. For more severe or persistent rash, temporary suspension of BTK-inhibitor therapy and use of topical or oral corticosteroids should be considered until resolution of cutaneous symptoms [10,43,49,50].

## 4. Vasculitis

Vasculitis is a group of diseases characterized by inflammation of the walls of blood vessels, which can lead to their narrowing, ischemia, and even bleeding. Diagnosis of ibrutinib-induced vasculitis is challenging, as the clinical manifestation of a specific vasculitic disorder depends on various factors, including the size and location of the involved vessels and the degree and pattern of extravascular inflammation [39].

Rodriguez-Baeza describes a patient with MCL receiving ibrutinib treatment who presented repeated episodes of lymphocytic vasculitis (LyV) [51]. Multiple erythematous papular lesions were observed to develop on the legs and arms several times, although each outbreak was followed by spontaneous resolution, without any need for treatment discontinuation. A biopsy of the lesion showed the presence of a dense infiltrate of lymphocytes, monocytes, and scattered eosinophils around small and medium-sized vessels on the superficial and deep dermis. Immunohistochemical staining confirmed the presence of a reactive CD3+ T cell infiltrate with a mixture of CD4+ and CD8+ lymphocytes and scattered CD20+ B lymphocytes; MCL was absent, as confirmed by CD5, SOX11, and cyclin D1 negativity.

Cutaneous leukocytoclastic vasculitis is clinically characterized by maculopapular skin rash caused by paraneoplastic dermatitis. The condition is characterized by the presence of perivascular inflammatory exudates with extravasation of red blood cells, together with elevated eosinophils, consistent with drug eruption [52,53]. Leukocytoclastic vasculitis is a very rare adverse event of ibrutinib treatment [44,54]. Kaya et al. describe leukocytoclastic vasculitis, manifested as multiple skin lesions, in a patient with CLL 13 days after ibrutinib initiation [55]. Patients with low-grade leukocytoclastic vasculitis-like eruptions may improve with topical corticosteroids and oral antihistaminic drugs. In more advanced symptoms, patients can be treated with oral corticosteroids with either BTKi dose reduction or cessation [43].

## 5. Neutrophilic Dermatoses

**Figure 4:**
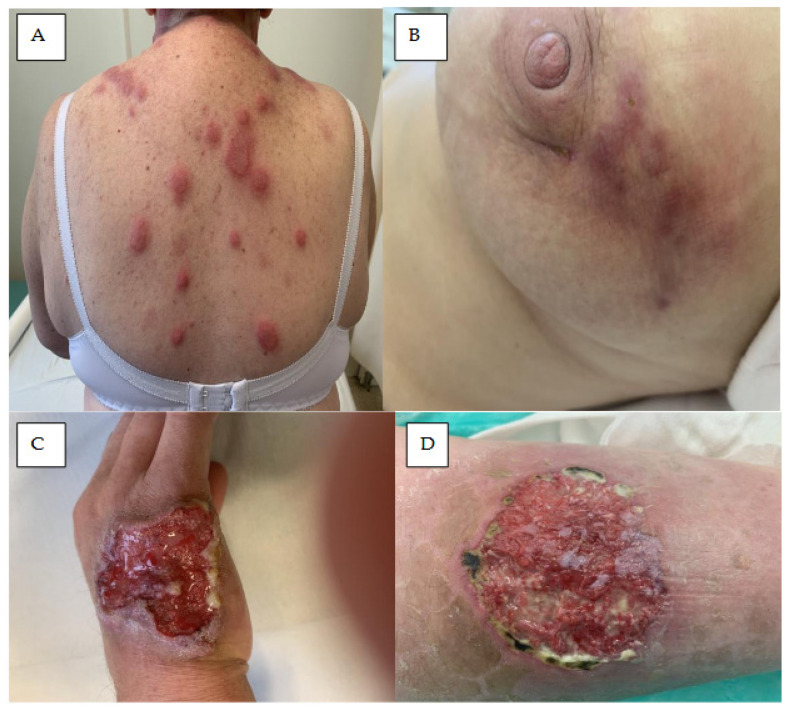
Neutrophilic dermatoses in patients treated with BTK inhibitors. (**A**) Sweet syndrome diagnosed two months after ibrutinib treatment initiation in a patient with CLL. Painful papules, nodules, and plaques accompanied by fever on the skin of the back. (**B**) Panniculitis neutrophilica. Tender red nodules on the breast skin in a patient with CLL treated for two months with acalabrutinib. (**C**,**D**) Pyoderma gangenosum. Rapidly spreading ulcers with raised edges on the skin of the right hand and lower leg in a patient with CLL treated with ibrutinib (**C**) and in the patients with MCL treated with acalabrutinib (**D**).

Neutrophilic dermatoses comprise a wide spectrum of diseases characterized by a dense infiltration composed mainly of neutrophils [56]. They are manifested by a constellation of clinical features, including fever, neutrophilic leucocytosis, raised painful plaques, and skin infiltration by neutrophils and include Sweet syndrome (Figure 4A), neutrophilic panniculitis (Figure 4B), and pyoderma gangrenosum (Figure 4C,D).

### 5.1. Acute Febrile Neutrophilic Dermatosis

Acute febrile neutrophilic dermatosis (Sweet syndrome) (Figure 4A) is a rare skin condition characterized by a sudden onset of painful, inflamed skin lesions [57,58].

The lesions may be few or numerous and may persist from days to weeks. They commonly affect the face, neck, and upper extremities. Hammel et al. describe a male CLL patient receiving ibrutinib who presented several migratory ecchymotic 2 to 3 cm nodules on the lower extremities [59]. A biopsy of a right leg nodule showed a mixed inflammatory panniculitis with small-vessel vasculitis and concomitant involvement of the septa and fibrinoid necrosis of vascular endothelium with associated neutrophils. The skin lesions completely resolved when ibrutinib was stopped, but new lesions developed within a week of recommencement. El Halabi et al. present a patient with CLL who developed several erythematous, painful, and papulonodular skin lesions in the limbs, neck, and face [60]. Neutrophilic dermatosis was confirmed by skin biopsy and Sweet syndrome was diagnosed. Ibrutinib therapy was stopped and the lesions disappeared; however, they returned following rechallenge with ibrutinib at full dose, indicating a direct relationship between dose and symptoms [60]. Another case with atypical neutrophilic dermatosis induced by ibrutinib was recently reported by Renuy et al. [61].

### 5.2. Neutrophilic Panniculitis

Neutrophilic (lobular) panniculitis (Figure 4B) is a very rare neutrophilic dermatosis characterized by subcutaneous nodular eruption and neutrophilic inflammation in subcutaneous fat. A few cases of neutrophilic panniculitis have been noted as rare cutaneous side effects of ibrutinib therapy [42].

In patients treated with ibrutinib, panniculitis has been described as painful erythematous nodules primarily involving the lower extremities and occurring within 1 to 90 days of starting treatment. They are characterized by septal and lobular inflammation. In most patients, ibrutinib discontinuation, or treatment with low-dose prednisone, was found to be effective [62,63].

Stewart et al. report a case with painful erythematous nodules on lower extremities which developed one month after initiating ibrutinib therapy for CLL [62]. Histological evaluation revealed lobular panniculitis with fat necrosis and infiltrates consisting predominantly of neutrophils. A diagnosis of neutrophilic panniculitis was rendered, which the patient opted not to treat. The lesions resolved spontaneously despite continued ibrutinib therapy [62].

Fabbro et al. reported five patients who developed panniculitis during ibrutinib therapy for lymphoid leukemia [42]. The patients presented with painful erythematous nodules at the extremities after initiation of ibrutinib therapy. Histopathological evaluation confirmed lymphohistiocytic, lobular panniculitis with prominent leukocytoclasis. Complete resolution of cutaneous lesions was observed when low-dose systemic corticosteroids were introduced, and nonsteroidal anti-inflammatory drugs in some patients.

### 5.3. Pyoderma Gangrenosum

Pyoderma gangrenosum (PG) is a rare, inflammatory, ulcerative dermatosis affecting mostly people between 20 and 50 years of age [64]. It may be caused by some drugs, including small molecules like tyrosine kinase inhibitors (TKIs) or BTK inhibitors [65,66]. It most commonly manifests as pustules or nodules, developing into a painful ulcer with undermined violaceous borders and a fibropurulent base (Figure 4C,D) [67,68]. The lesions may be single or multiple, and the most prevalent locations are the lower limbs, followed by the trunk; it can also manifest as arthritis or a hematologic disorder.

Sławińska et al. describe the case of a patient with CLL who developed PG six months after initiation of ibrutinib therapy (3 × 140 mg/d) [65]. Ibrutinib was discontinued and the patient treated with prednisone and cyclosporin A. The patient recovered with healing being almost complete after six weeks. Similarly, Giovanni et al. report the case of another patient with CLL who developed PG after five months of ibrutinib treatment [67]. Subsequent steroid treatment resulted in visible improvement; while remission could only be maintained with continued low-dose prednisone, the lesions disappeared completely when ibrutinib was switched to venetoclax. Finally, Pinato et al. report fifteen cases of PG, including eight cases associated with sunitinib, two with imatinib, two with ibrutinib, one with gefitinib, one with pazopanib, and one with dabrafenib and trametinib [69].

## 6. Skin Infections

**Figure 5:**
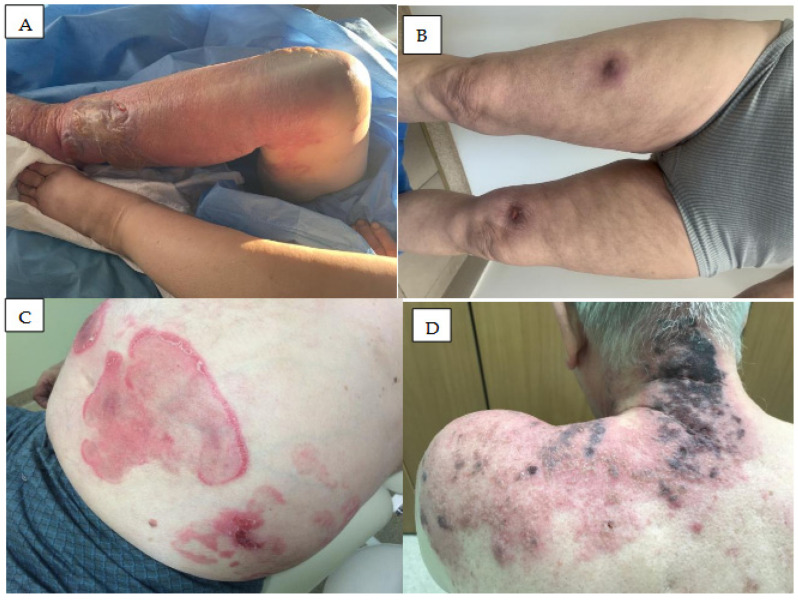
Skin infections in patients treated with BTK inhibitors. (**A**) Erysipelas on the right lower legs in a patient with CLL on the fifth week of treatment with ibrutinib. (**B**) Disseminated boils on both thighs in a patient with CLL in the second year of zanubrutinib treatment. (**C**) Fungal infection on the trunk of a patient with MCL six months after starting ibrutinib treatment. Peripherally spreading erythematous scaly lesions with the presence of exudative papules on periphery and accompanying itching. (**D**) Herpes zoster on the neck and left sideburn in a patient with CLL treated for six months with ibrutinib. Erythematous vesicular lesions with a necrotic component.

Treatment with BTK inhibitors weakens the immune system; as such, patients are at an increased risk of various infectious complications, including skin infections [20,70,71]. Skin infections by bacteria, fungi, viruses, or parasites can cause cutaneous symptoms such as rashes, swelling, itching, and pain (Figure 5) [72]. The most common bacterial infections are associated with *Staphylococcus aureus*, typically as moderate folliculitis [12,17,73]. A systematic review of 48 prospective trials of hematologic malignancies by Tillman et al. classified the occurrence of infectious AEs [74]. Infections of any grade were noted in 56% of patients treated with single-agent ibrutinib and 52% of those receiving combination therapy. Grade 3-4 infectious AEs occurred in 26% (single-agent ibrutinib) and 20% (combination). In both groups, the rate of grade 5 infectious AEs was 2% [74].

In another study of 378 patients treated with ibrutinib over a 5-year period, serious infections were observed in 11%, with most arising during the first year of ibrutinib therapy [75]. Importantly, an increased risk of opportunistic infections was noted, especially by fungal infections, most commonly *Aspergillus* spp. [76,77]. Singh et al. describe a patient with CLL who developed skin toxicity in the perianal area during ibrutinib treatment, leading to a superimposed bacterial infection and perianal cellulitis. As a result, ibrutinib was discontinued and antibiotic treatment initiated [70].

Opportunistic skin infections, mainly due to non-tuberculous mycobacteria or mucormycosis, have also been associated with inibrutinib treatment [78,79]. An increased risk of herpes virus infection was also reported in patients treated with ibrutinib and acalabrutinib [73,80,81,82,83]. Prophylactic use of valaciclovir or Herpes zoster vaccination should be considered in patients with common Herpes infection before treatment with BTK inhibitors.

## 7. Mucosal Symptoms

**Figure 6:**
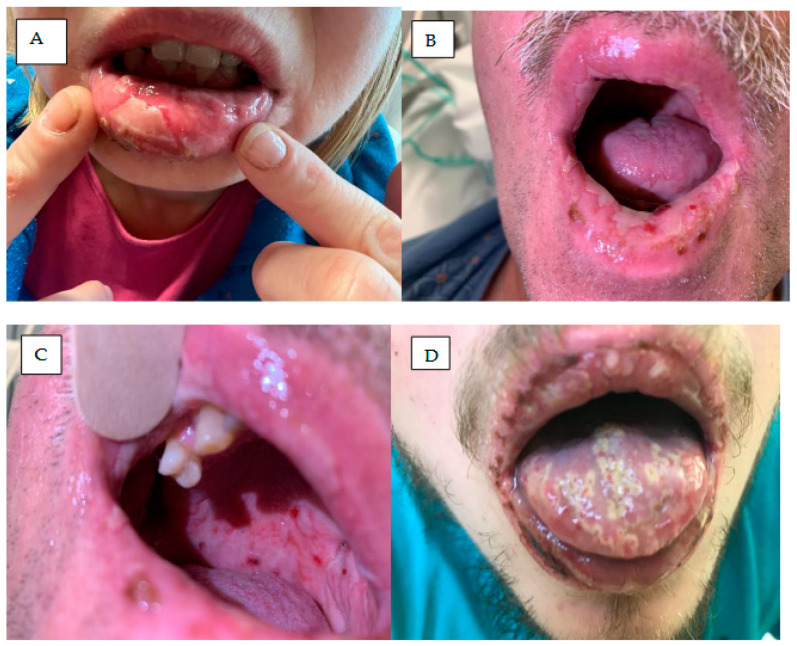
Mucosal symptoms in a patient with MCL treated for two weeks with acalabrutinib. (**A**,**B**) Extensive oral mucositis with painful, diffused erosions and necrotic ulcers with fibrin present. Circumscribed necrotic ulcer with erythematous halo located on lip mucosa partly covered by a yellow fibrin. (**C**) Multiple hemorrhagic lesions located on mucosa of plate and lower lip. (**D**) Mucosal lesions located on tongue, mimicking major aphthous stomatitis in the same patient.

Mucosal symptoms have been described in around 10% of patients treated with ibrutinib, but only 1–3% were grade 3 or higher [12]. In a pivotal study performed in treatment-naïve (TN) CLL patients, all-grade mucositis was reported in 11% of patients and high-grade stomatitis in 1%; however, the clinical aspects were not described [84,85,86]. Clinically, mucosal changes manifest as painful oral necrotic ulcers mimicking aphthous stomatitis. Ibrutinib can also induce severe impairing stomatitis (Figure 6).

Vigarios et al. describe three patients treated with ibrutinib who developed a grade ≥ 3 stomatitis [85]. Two patients reported painful necrotic aphthous-like ulcers that developed within 4 weeks or even 16 months from the beginning of treatment. In three patients, the resulting skin changes required treatment interruption. in all three patients, the mucosal lesions resolved within a week of treatment discontinuation combined with supportive care including basic oral or steroid care. Reintroduction of treatment at a lower dose did not result in any recurrence of the lesions [85]. In general, for patients treated with BTKi, oral care can prevent infection, control pain, improve quality of life, and manage concomitant oral complications [87].

## 8. Nail Lesions and Hair Abnormalities

**Figure 7:**
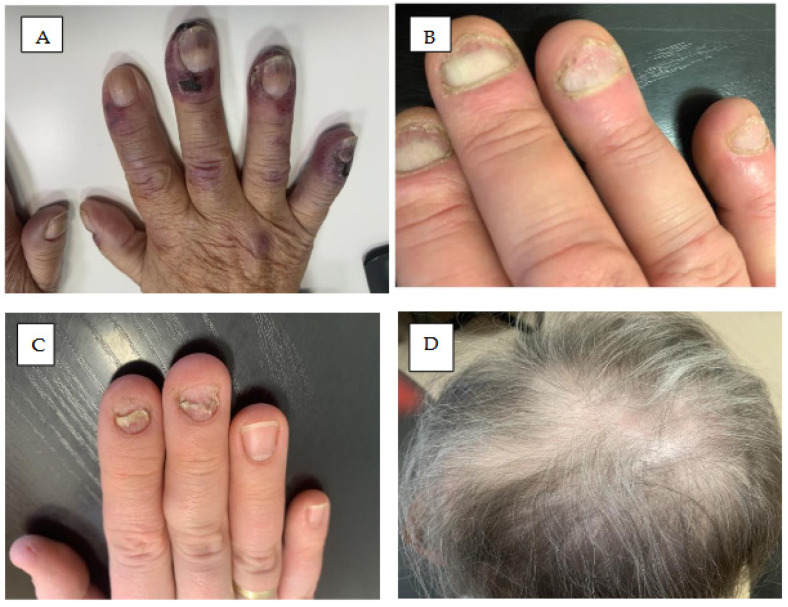
Nail lesions and hair abnormalities in patients treated with ibrutinib. (**A**) Hemorrhagic and necrotic ulcers on the skin and nail plates of the right hand of a patient treated for two months with ibrutinib. (**B**) Excessive drying, delamination, and chipping of fingernails in CLL patient six months after treatment with ibrutinib. (**C**) Onychodystrophy of the nails of the second and third fingers of the right hand in a patient with MCL treated for six months with ibrutinib. (**D**) Diffuse hair loss with noticeable thinning on the top of the head in a CLL patient after one-year treatment with ibrutinib.

Hair and nail abnormalities are commonly associated with ibrutinib treatment and usually appear several months after treatment initiation (Figure 7) [88,89].

### 8.1. Nail Changes

Patients treated with ibrutinib often demonstrate nail changes, such as brittleness onycholysis, onychorrhexis, onychoschizia, koilonychia, trachyonychia, paronychia, and subungual splinter hemorrhages (Figure 7A–C). The most common symptoms are mild to moderate nail cracking and detachment, onychoschizia, and onychorrhexis; however, these are mainly grade 1 or 2 severity and do not require treatment [22,90]. Nail changes are typically located on the fingers, develop gradually, and are diagnosed after several months of treatment [88,89,91].

Ibrutinib-induced pyogenic granuloma, a benign vascular tumour also known as lobular capillary hemangioma, has also been observed [91]. It manifests as a solitary and rapidly growing papule or nodule. In some patients, fragile and brittle nails with trachyonychia, linear splitting, horizontal splitting, and shedding of proximal nails (onychomadesis) have been reported, mainly on the fingers [88,89,91].

Farooqui et al. report nail ridging in 22 of 51 (43%) CLL patients treated with ibrutinib [92]. Another study of 66 patients with CLL treated with ibrutinib found 44 (67%) developed brittle fingernails after a median of 6.5 months and 15 (23%) brittle toenails after a median of 9 months [88]. The mechanism of drug-induced alterations in nail texture is multifactorial and may be related to its influence on cysteines, which are critical for nail hardness: ibrutinib may increase nail brittleness by disrupting the disulphide bonds between cysteine residues. Ibrutinib-related nail plate abnormality is not a drug-limiting toxicity in most patients [89].

A study of 66 patients undergoing long-term treatment with ibrutinib found 67% to have experienced fingernail brittleness and 22.7% toenail brittleness. These changes manifested over the course of six to nine months of treatment [88].

In most patients, nail changes do not represent a dose-limiting toxicity and no treatment is needed. Nevertheless, patients with nail changes should keep their nails short and polish them once a week. Daily biotin can also be useful [89].

### 8.2. Hair Changes

Around 20–30% of patients treated with ibrutinib demonstrate hair changes [88,92] such as straightening and softening, increased curliness, and alopecia, with the hair follicle changing from curly to straight (Figure 7D) [88]. Ibrutinib may have a similar influence on the disulphide bonds in hair as those in nails: a scanning electron microscopy study confirmed changes in the hair shaft, similar to those following EGF inhibitors [93]. In some patients, hair changes can be minimized by minoxidil treatment [86].

## 9. Conclusions

BTK inhibitor therapy is associated with a number of side effects, including bruising, rashes, hair changes and nail plate abnormalities, and less commonly, purpuric painful nodules and pyoderma gangrenosum. They are most closely associated with ibrutinib treatment, but also result from acalabrutinib or zanubrutinib treatment. However, patients typically experience longer exposure to ibrutinib than acalabrutinib or zanubrutinib: a head-to-head comparison found ibrutinib to be associated with a similar frequency of skin changes as acalabrutinib or zanubrutinib and a similar frequency of bleeding events as patients treated with pirtobrutinib.

For most patients, the skin changes are self-limiting and they can be relieved with local symptomatic treatment or be managed with local or systemic steroids. However, BTKi dose reduction, treatment interruption, or even complete cessation is recommended.

## Data Availability

The original contributions presented in this study are included in the article. Further inquiries can be directed to the corresponding authors.
